# Preparation and Performance of Silica-di-Block Polymer Hybrids for BSA-Resistance Coatings

**DOI:** 10.3390/ma13163478

**Published:** 2020-08-07

**Authors:** Hongpu Huang, Yefeng Feng, Jia Qu

**Affiliations:** 1 School of Materials Science and Engineering, Yangtze Normal University, Chongqing 408100, China; feng_ye_feng@126.com; 2 Shaanxi Key Laboratory of Comprehensive Utilization of Tailings Resources, Shaanxi Engineering Research Center for Mineral Resources Clean & Efficient Conversion and New Materials, Shangluo University, Shangluo 726000, Shaanxi, China; qj0424@126.com

**Keywords:** silica-di-block hybrids, LCST, surface properties, BSA resistance, coating

## Abstract

A series of tem-responsive and protein-resistance property silica-di-block polymers SiO_2_-g-PMMA-b-P(PEGMA) hybrids are synthesized with methyl methacrylate (MMA) and poly (ethylene glycol) methyl ether methacrylate (PEGMA) by the surface-initiated atom transfer radical polymerization (SI-ATRP). The morphology in tetrahydrofuran (THF) solution, lower critical solution temperature (LCST), surface morphology, bovine serum albumin (BSA)-resistance property, and thermal stability of nanoparticles were analyzed. The results of ^1^H-NMR, GPC, and TEM prove that the silica-di-block hybrids have been obtained. The silica-di-block hybrids shows the LCST (52–64 °C) in aqueous solution. The hybrid films casted by THF present distributed uniform granular bulges and the film surface is relatively smooth (Ra = 15.4 nm ~ 10.5 nm). The results of QCM-D showed that only a small amount of BSA protein(△*f* = 18.6 ~ 11.8 Hz) was adsorbed on the surface of the films. The result of XPS also demonstrated that only a small amount of BSA protein was absorbed onto the surface of the film (N% = 1.86). The TGA analyses indicate that the thermal decomposition temperature of hybrids is 288 °C. Thus, it is suggested that the hybrids are served as a suitable coating with BSA resistance property and thermal stability.

## 1. Introduction

Nanohybrid materials formed by inorganic nanoparticles and polymers have become the main methods to improve the properties of polymers and provide an effective way to prepare new coating materials [1,2]. Silica/polymer hybrid material graft block copolymer on nano-silica surface combines dual properties of silicon dioxide and block polymer, showing broad application prospects in biomedicine [3,4], antibacterial coating [5,6,7], colloid science [8,9], and advanced functional materials [10,11]. Silica particles have high specific surface area, easy preparation, particle size, easy to control, and easy to the characteristics of surface functionalization [12,13] and have an important role in preparing silica/polymer hybrid materials. Silica/polymer hybrid materials are widely investigated as functional coating materials [10,14]. The uniform dispersion of silica in the polymer matrix is the key point to control the properties of the materials. An effective way to avoid agglomeration between particles is by chemically grafting polymers onto the silica particles. This method could improve the dispersion stability of silica particles and the compatibility of silica particles with polymer matrix. It is an effective method to graft different types of polymerization on the surface of silica using controlled/active radical polymerization [15,16,17]. The structure of the polymer can be skillfully controlled by adjusting the graft density, composition, and feed ratio of the polymer on particle surface [17]. The silica/polymer hybrids are widely used in the fields of hydrophobic coatings [18], biological materials [19], protein-resistance coatings [20], and antioxidant hybrid materials [21,22]. Typically, the SI-ATRP approach is one common active controlled radical polymerization technique to obtain silica/polymer hybrid materials [16]. Among various silica/polymer hybrids, coatings with protein resistance property attracted much attention of researchers [23,24]. Coatings with protein resistance property can prevent microorganisms from attaching to the surface [25]. PEG-based materials show protein resistance property via steric repulsion and surface hydration. Andrade and de Gennes [26,27] studied the adsorption resistance of proteins on the functionalized surface of polyethylene glycol (PEG) and it was found that a long, hydrated polymer chain creates steric repulsion to prevent protein absorption when proteins approached. Two of the most commonly used methods for quantitative characterization of the amount of adsorbed protein are surface plasmon resonance (SPR) [28] based on optical principle and quartz crystal microbalance (QCM-D) [29] based on acoustic principle. Based on the real-time monitoring and high sensitivity, QCM-D is widely used to detect the adsorption of proteins and other biological macromolecules on the surface [30]. The protein resistance property of polymer hybrids can be regulated by regulating the block composition of the polymer. The PEG-based materials to improve their protein resistance performance has been extensively studied [31,32,33,34,35]. For instance, recent work successfully prepared PEGylated poly (ether imide) (PEI) ultrafiltration membranes by surface modification [31]. The obtained PEGylated PEI membranes provided excellent resistance to the protein fouling. For the same purpose, the PEGylated copolymers of poly (ethylene glycol) methacrylate (PEGMA) and polystyrene (PS) [36] were prepared by atom transfer radical polymerization. Then, the PEGylated copolymers were anchored on the PVDF membrane. The obtained PEGylated PVDF- membrane had excellent resistance to bovine serum albumin (BSA).

PEGMA is composed of reactive methacrylate main chain and hydrophilic polyethylene glycol side chain (PEG) [37] and can be used to prepare various protein resistance materials through active controllable polymerization technology [38]. PEGMA-based materials have hydrophilic side chain PEG, which can form a hydrated layer on materials surfaces by hydrogen bonding. The hydrated layer could generate a steric repulsion to prevent protein absorption. Therefore, PEGMA-based coating materials have potential applications in protein-resistant materials [39,40]. For instance, the amphiphilic ternary copolymers P(H−P−A) were synthesized by free-radical polymerization [41]. It has been found that the polymer film has excellent antibacterial and anti-protein properties. Although surface grafting polyethylene glycol (PEG) is one of the most widely used methods to prepare materials with protein-resistance property, it has some drawbacks in film-forming property. On the other hand, the acrylate polymers have remarkable film-forming performance, so it can be designed as a block segment to film-forming performance and excellent surface properties [42,43]. One approach to solve this problem is by introducing block polymers with excellent film-forming property to the PEG-based copolymers, which could combine both protein-resistance property and film forming property.

In our previous work, we have reported on the preparation of silica/polymer coating with protein resistance properties by SI-ATRP [44]. The coating shows an adequate hydrophilic and BSA resistance property. The incorporation of hydrophilic polymers can still enhance BSA resistance property which would cause reduced film-forming performance. In this study, we introduced methyl methacrylate (MMA) to improve the film-forming performance of the hybrid film and obtain coatings with an adequate film-forming property, thermal stability, and BSA resistance. The silica di-block polymer hybrids were prepared by MMA and PEGMA via the SI-ATRP. The chemical structures for hybrids are characterized by nuclear magnetic resonance (^1^H-NMR) spectroscopy and gel permeation chromatography (GPC) analyses. The obtained hybrids in tetrahydrofuran (THF) solution are characterized by transmission electron microscopy (TEM). The transition point for the lower critical solution temperature (LCST) is examined by dynamic light scattering (DLS). The surface properties of hybrid films are investigated by atomic force microscope (AFM) and static contact angle (SCA). The BSA resistance property of films is investigated by the quartz crystal microbalance with dissipation (QCM-D) and XPS. The thermal stability of hybrids is investigated by TGA analyses. 

## 2. Materials and Methods 

### 2.1. Materials

The materials are shown as follows. Silica nanoparticle (SiO_2_) using fumed silica (VK-SP15) with an average diameter of 10–25 nm and a specific area of 230 m^2^/g was supplied by Hang Zhou New Material Company of China (Hangzhou, China). The silica-initiator (SiO_2_-Br) was prepared using the previous method [45]. MMA (99 wt%, Sigma Aldrich (Shanghai) Trading Co., LTD, Shanghai, China) was purified by extraction with 5% aqueous sodium hydroxide and distilled from calcium hydride. Poly (ethylene glycol) methyl ether methacrylate (PEGMA, ~475 g·mol^−1^) was supplied by Aladdin Industrial Corporation (Shanghai Alighting Biochemical Technology Co., LTD, Shanghai, China). Bovine serum albumin (BSA) solution was prepared in distilled water and phosphate buffer solution (PBS) with a pH of about 7.4 at 20 °C. N, N-dimethylformamide (DMF) was stirred over CaH_2_ for 12 h at room temperature, and then distilled under reduced pressure prior to use. CuCl and CuCl_2_ were purified as previous method [45]. PMDETA, THF, and other solvents were used directly.

### 2.2. Preparation of SiO_2_-g-PMMA-b-P(PEGMA)

#### 2.2.1. Preparation of SiO_2_-g-PMMA

**Scheme 1:**
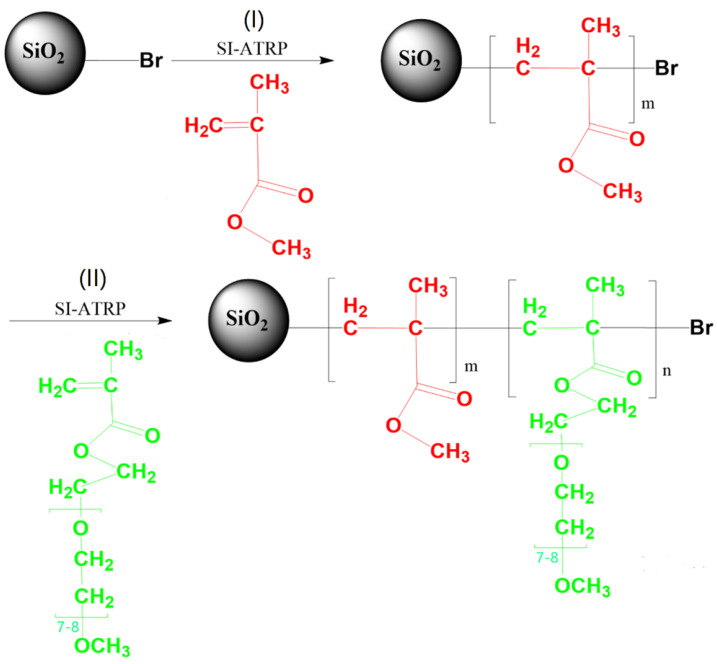
The synthesis route of SiO_2_-g-PMMA-b-P(PEGMA).

The synthesis route for SiO_2_-g-PMMA is shown in Scheme 1. Under N_2_ atmosphere, 0.5626 mmol CuCl, 0.0443 mmol CuCl_2_, 56.26 mmol MMA, and 0.5626 mmol PMDETA were added into a 100 mL dried Schlenk flask, which is sealed with a rubber septum prior to three vacuum/N_2_ cycles. When the mixture is evenly stirred, 10 mL SiO_2_-initiator (0.5626 mmol in DMF) was added to the flask by cannula transfer. Then, the reaction proceeds at 90 °C and the reaction continued for 12 h. When the polymerization stopped, the mixture was diluted by THF. Then, the hybrids were isolated by centrifugation. The obtained particles were repeatedly dispersed in THF and isolated by centrifugation. The process was repeated four times to remove physically adsorbed polymers. The obtained SiO_2_-g-PMMA was dried in a vacuum oven at 40 °C for 12 h. The conversion rate of MMA is about 82%. 

#### 2.2.2. Preparation of SiO_2_-g-PMMA-b-P(PEGMA)

The P(PEGMA) blocks were grafted onto SiO_2_-g-PMMA to obtain SiO_2_-g-PMMA-b-P(PEGMA) via a second ATRP step, as shown in Scheme 1(II). The reaction was taken under 90 °C and continued for 12 h. The conversion rate of PEGMA after 12 h reaction is about 80%. We obtained three kinds of polymers with molar ratios as SiO_2_-Br/MMA/PEGMA = 1/100/18, 1/100/32 and 1/100/52 as Sample S1, S2, and S3. The polymerization condition and detailed ingredient ratio are listed in Table 1.

### 2.3. Characterization of Hybrids

#### 2.3.1. The Chemical Structure

The chemical structure for SiO_2_-g-PMMA and SiO_2_-g-PMMA-b-P(PEGMA) was characterized by nuclear magnetic resonance (^1^H NMR) spectroscopy (BrukerAC500 NMR spectrometer, Bruker, Karlsruhe, Germany) and deuterium generation of chloroform was used as solvent. The molecular weights of the PMMA-b-P(PEGMA) cleaved from SiO_2_-g-PMMA-b-P(PEGMA) were measured by gel permeation chromatography (GPC, Wyatt DAWN EOS MZ 10^3^+MZ 10^4^) at 25 °C. DMF was used as the eluent and the flow rate is 0.5 mL·min^−1^.

#### 2.3.2. The Morphology 

The morphology of obtained SiO_2_-g-PMMA-b-P(PEGMA) hybrids in THF solution were observed by transmission electron microscopy (TEM) with a JEM-3010 TEM (JEOL, Tokyo, Japan) at an acceleration voltage of 100 kV. 

#### 2.3.3. The Surface Properties 

The SiO_2_-g-PMMA-b-P(PEGMA) films were prepared by casting 10 wt% THF solution on glass slide and dried at ambient temperature for 72 h. The surface morphology of films was characterized by atomic force microscope (AFM, Bruker, Karlsruhe, Germany). The measurements of AFM were taken at room temperature under 38–42% R.H. Tip information was <10 nm radius, 40 ± 3 mm width, 2.0 ± 0.5 mm thickness, 90 ± 5 mm cantilever length, 330 kHz resonant frequency, and 48 N/m force constant. The contact angle of water of the film is determined by static contact angle (SCA) at 25 °C and ±0.5° instrument error.

#### 2.3.4. The Lower Critical Solution Temperature (LCST) 

LCST of samples in water was characterized by dynamic light scattering (DLS) (MALVERN Nano ZS 90, Malvern Panalytical, Marvin, UK). The LCST of samples was determined at a heating rate of 0.4 °C·min^−1^ with an interval of 2.0 °C. Each measured temperature point is kept for three minutes before measurement for equilibration. The samples were diluted in water (1.0 mg·mL^−1^) to prevent aggregation.

#### 2.3.5. Thermal Stability of Hybrids 

The thermal stabilities of the obtained hybrids were characterized by thermo gravimetric analysis (TGA) (STA449C Jupiter from NETZSCH, Bavaria, Germany). The test was performed under N_2_ atmosphere and the rate of temperature is 10 °C·min^−1^ until to 800 °C.

### 2.4. BSA Adsorption Measurement 

The BSA resistance property of sample films was monitored by Quartz crystal microbalance with dissipation (QCM-D) (Q-Sense E4, Sweden Baolin Technology Co., LTD, Gothenburg, Sweden) at 25 °C. AT-cut piezoelectric quartz crystals covered with gold were used with a fundamental frequency of 5 MHz and a diameter of 14 mm. Sample solution with 0.5 mL was dropped on the surface of quartz crystal and drying in a vacuum oven at 40 °C for 24 h to prepare film sample. BSA was dissolved in 0.01mol·L^−1^ buffer solution (PBS, pH 7.4) at a concentration of 0.4 g/L. During the test, PBS solution of 0.01 mol·L^−1^ was first added until steady as the baseline, then BSA solution was added until adsorption equilibrium was reached about 60 min, and then PBS solution was added for 40 min to wash the BSA. The change in frequency (Δf) and energy dissipation (ΔD) were recorded at 15 MHz during the adsorption process. 

The BSA-resistance property of sample films was also evaluated by X-ray photoelectron spectroscopy (XPS) (AXIS ULTRA, Manchester, England, KRATOSANALYTICAL Ltd) with an Al-Ka monochromatic X-ray source (1486.6 eV) operated at 150 W. The overview scans were tested with a pass energy of 160 eV and acquisition time of 220 s.

In order to improve the readability of the manuscript, all original names and abbreviations are listed in Table 2.

## 3. Results and Discussion

### 3.1. The Chemical Structure of Hybrids

**Figure 1:**
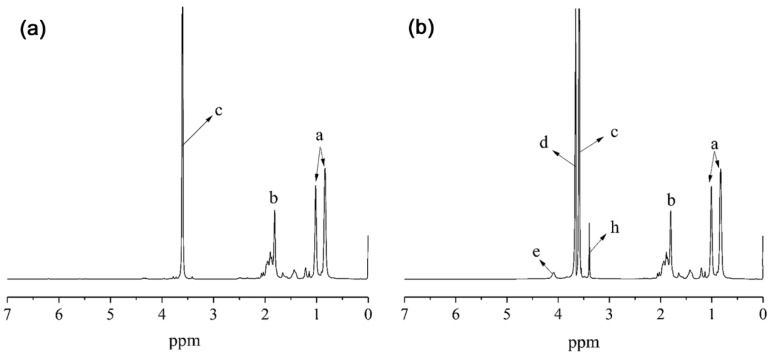
Nuclear magnetic resonance (^1^H-NMR) spectra of SiO_2_-g-PMMA (**a**) and SiO_2_-g-PMMA-b-P(PEGMA) (**b**).

To confirm the chemical structure of hybrids, ^1^H-NMR was performed. The ^1^H-NMR spectrum of SiO_2_-g-PMMA and SiO_2_-g-PMMA-b-P(PEGMA) are shown in Figure 1a,b. The signals of -C-CH_3_ and (-CH_2_-CH_2_-) protons in the backbone of methacrylate of PMMA and P(PEGMA) were at δ 0.841 and 1.020 ppm (peak a) and 1.83~2.08 ppm (peak b). The characteristic signals for the -O-CH_3_ group of PMMA was at 3.600 ppm (peak c). The characteristic peak for methylene (-OCH_2_-CH_2_-) of P(PEGMA) was at δ 3.38 ppm (peak h). The characteristic peak for methoxy (-OCH_3_) of P(PEGMA) was at δ 3.65 ppm (peak d). The signal for -CH_2_ of the PEG next to -O-C=O was found at δ 4.21 ppm (peak e). The characteristic peaks of both SiO_2_-g-PMMA and SiO_2_-g-PMMA-b-P(PEGMA) can be seen in the Figure 1a,b and it is proved that SiO_2_-g-PMMA-b-P(PEGMA) is prepared by SI-ATRP. 

**Figure 2:**
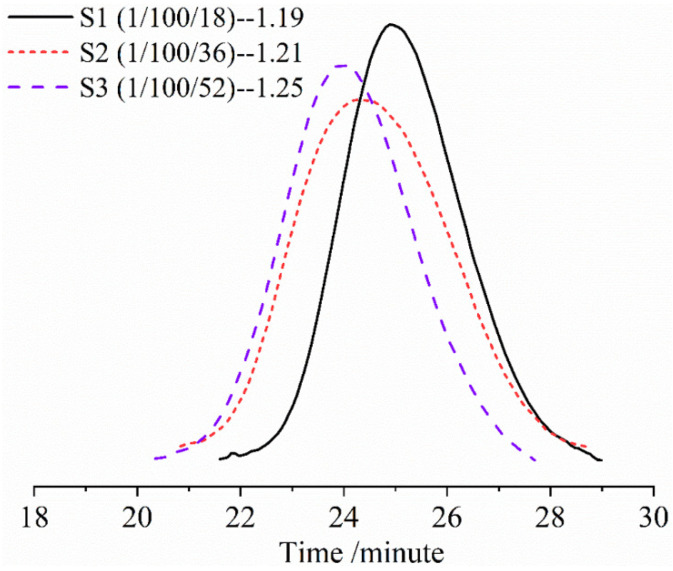
Gel permeation chromatography (GPC) curves of the PMMA-b- P(PEGMA) cleaved from SiO_2_-g-PMMA-b-P(PEGMA) in S1, S2, and S3.

The molecular weights of SiO_2_-g-PMMA-b-P(PEGMA) for S1, S2, and S3 are shown in Figure 2 and Table 3. The numeral average molecular weights of S1, S2, and S3 were 15,800, 21,800, and 26,600 g·mol^−1^, respectively. In Figure 2, the polydispersity index was shown as PDI = 1.19–1.25 and this indicates that the molecular weight of hybrids has a narrow distribution, which illustrate that it shows that the polymerization process of SI-ATRP belongs to controllable polymerization. At the same time, the molecular weight increases with the increase of moles of monomer added from sample 1 to sample 3, which proves that the polymers have been grafted onto the silica particles. The results of ^1^H-NMR and GPC can prove that the SiO_2_-g-PMMA-b-P(PEGMA) hybrids have been obtained as expected.

### 3.2. The Morphology of SiO_2_-g-PMMA-b-P(PEGMA) Hybrids 

**Figure 3:**
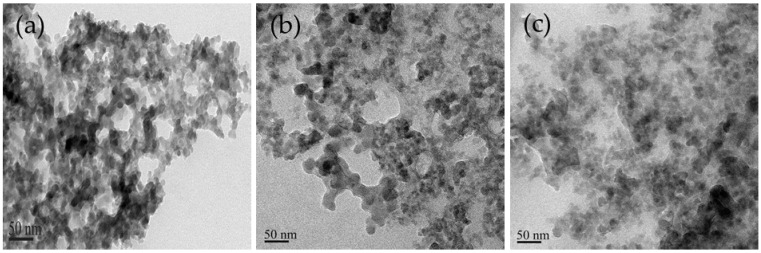
Transmission electron microscopy (TEM) results of hybrids for (**a**) S1, (**b**) S2, and (**c**) S3 in tetrahydrofuran (THF) solution.

The surface performances of films could be affected by the self-assembly aggregates of hybrids. For studying the influence of different mass ration of hybrids on the morphology, we studied the aggregation patterns of the hybrids in THF solution by TEM. The images of the hybrids are shown in Figure 3.

The images of S1, S2, and S3 by TEM in THF solution are shown in Figure 3a–c. All the hybrid particles present a granular distribution and the particles are interconnected. The silica is located at the core of the particles, and the light outer layer of the particles and the light parts between the particles should be polymer. At the same time, it can be seen from the Figure 3a–c that the particles are evenly distributed, but there is still an amount of agglomeration. As the content of P(PEGMA) increases from S1 to S3, the particles were more interconnected coherently with less agglomeration. This result suggests that more polymers were grafted onto silica and also proves that the SiO_2_-g-PMMA-b-P(PEGMA) hybrids have been obtained.

### 3.3. The LCST of Hybrids 

**Figure 4:**
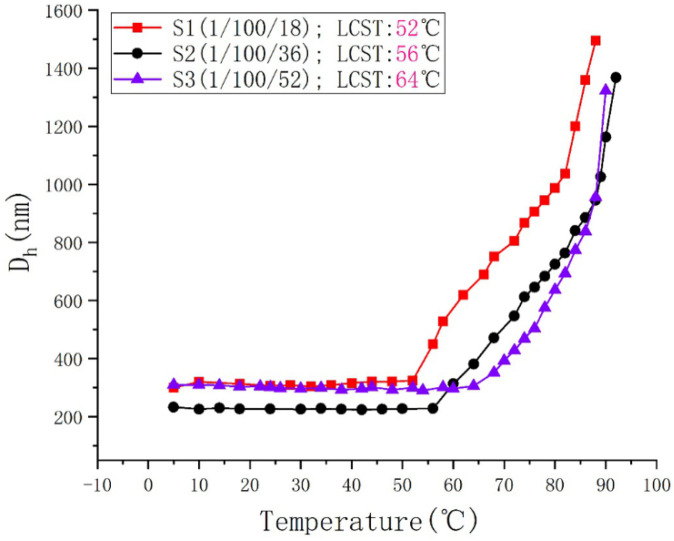
Dynamic light scattering (DLS) results of SiO_2_-g-PMMA-b-P(PEGMA) hybrids in aqueous solution.

Based on PEGMA’s temperature sensitivity [37], the particle size of SiO_2_-g-PMMA-b-P(PEGMA) hybrids in aqueous solution was measured by DLS at different temperature, and then the curve of particle size with temperature was made. The test concentration is 1.0 mg/mL. The temperature corresponding to the transition point of sudden increase of particle size in the graph is LCST. The LCST graph of S1, S2, and S3 is shown in Figure 4 and the LSCT of S1, S2, and S3 is 52 °C, 56 °C, and 64 °C respectively. However, the LCST temperature of pure P(PEGMA) is 90 °C [39], this indicates that the introduction of silica and hydrophobic PMMA blocks can significantly reduce LCST temperature. From S1 to S3, LCST temperature increased with the growth of hydrophilic chain P(PEGMA) segments. This is because the increase of hydrophilic P(PEGMA) chain segments will weaken the hydrophobicity of hydrophobic PMMA blocks, thus increasing the hydration of hybrids and weakening the molecular force between hybrids, thus the LCST is increased. In addition, during the phase transition, the water loss process of nanoparticles can be obtained from the curve. For all samples, the sudden increase of particle size at the turning point indicates that the separation process of hybrids and water molecules is relatively rapid. This causes the particles to aggregate together, so that the particle size will suddenly turn larger. When the temperature is higher than LCST, P(PEGMA) becomes hydrophobic, and the molecular force between hybrids is gradually enhanced, resulting in aggregation and formation of precipitation.

### 3.4. Surface Morphology and the Protein Resistance of Films

**Figure 5:**
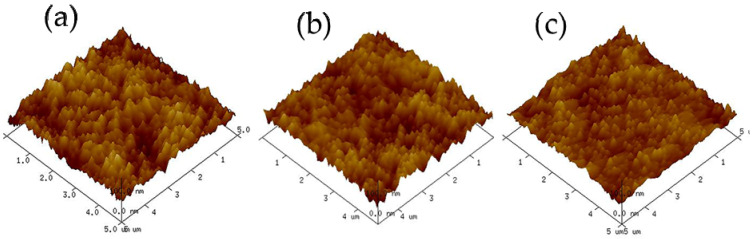
Atomic force microscope (AFM) results of films for (**a**) S1, (**b**) S2, and (**c**) S3 casting by THF.

AFM was used to investigate the morphology of hybrid films. In Figure 5a–c, the films of S1, S2, and S3 all present some granular islands. From S1 to S3, the height of the granular islands on the film surface are dropped because of the increase of P(PEGMA) and molecular weight. Meanwhile, the roughness (Ra) of films is decreased (Ra = 31.3 nm, 29.7 nm, and 28.9 nm, respectively) from S1 to S3. As the content of P(PEGMA) increases from S1 to S3, the PEG side chains tend to be on the surface of the films during film formation. Thus, the film surface formed is relatively smooth and the surface roughness gradually decreased. All sample films show a relatively smooth surface because of the enough dispersion of hybrids in THF solution. 

Actually, the film surface roughness has an effect on the film surface wettability. Therefore, we measured the SCA of the films with the static contact angles (SCAs) measurements. The SCA of films is shown in Table 4. With the decrease of surface roughness from Sample S1 to S3 (Figure 5), the surface wettability is improved. It is well-known that PEGMA has hydrophilic property because of its PEG side and PMMA has weak hydrophobic property. Therefore, SiO_2_-g-PMMA-b-P(PEGMA) films show an adequate hydrophilic property. The Sample 1 shows highest static contact angle (35°) because it has the highest hydrophobic PMMA content. As the Sample 3 has the highest hydrophilic P(PEGMA) content, the water spreads out on the film surface and the water contact angle is not detected.

**Figure 6:**
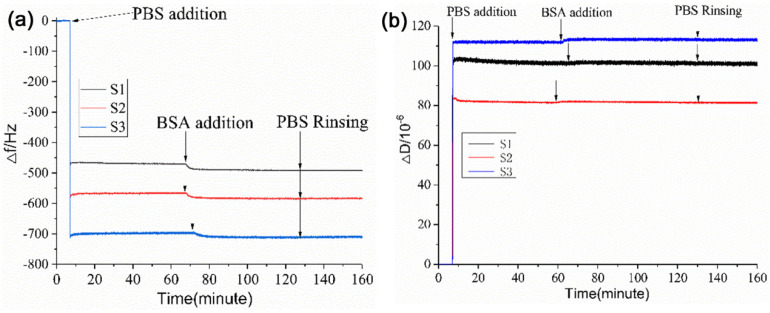
Quartz crystal microbalance with dissipation (QCM-D) results of S1, S2, and S3 films casted by THF solution. (**a**) △*f*; (**b**) △*D*.

PEG-modified surfaces are often used as protein resistance materials [46]. PEG-coated surfaces are prone to hydration and create a steric repulsion because of a low interfacial energy with water, and their protein resistance depends on the steric repulsion. The protein resistance ability of the membrane depends on the protein adsorbed on the film surface and the less protein adsorbed, the better protein resistance ability. Thus, the BSA resistance properties of SiO_2_-g-PMMA-b-P(PEGMA) films were characterized by using QCM-D technology [37] and the results of QCM-D are shown in Figure 6. First, 0.01mol·L^−1^ PBS buffer was added. After the adsorption reached equilibrium, 0.4g·L^−1^ BSA buffer was added. After the BSA adsorption reached equilibrium, PBS buffer was added for rinsing. The △D indicated the viscoelasticity of the film and the △f indicated the amounts of adsorbed liquids on the film surface. In Figure 6a, the △*f* of S1–S3 decreased slightly(△f = −18.6 Hz, −15.4 Hz and −11.6 Hz, respectively) when BSA solution was added as shown in Table 5, which indicates that a little adsorbed BSA protein was on the film surface. Meanwhile, △D from S1 to S3 films only slightly increased (Table 3), further indicating that a little adsorbed BSA protein was on the film surface. QCMD result proves that the films had excellent protein-resistance property. This is because the P(PEGMA) segments have excellent hydrophilicity and PEG side chains are distributed on the film surface during film formation. As the protein solution passes over the film surface, the PEG side chains hydrate with the water to form a hydration layer, which could generate a repulsive force on protein adsorption. At the same time, the terminally attached PEO chains are compressed because of the steric hindrance effect. Steric resistance and intermolecular forces exist between solid substrates and proteins, but the intermolecular forces is small in comparison with the steric repulsion. Therefore, the hydration layer and the steric hindrance effect of PEG side chains make the films have an adequate BSA resistance property. At the same time, it can be found that from S1 to S3 with the growth of P(PEGMA) content, the absorbed BSA protein amount (△*f*) decreases, indicating that the content of P(PEGMA) increased, and its BSA resistance performance is improved.

**Figure 7:**
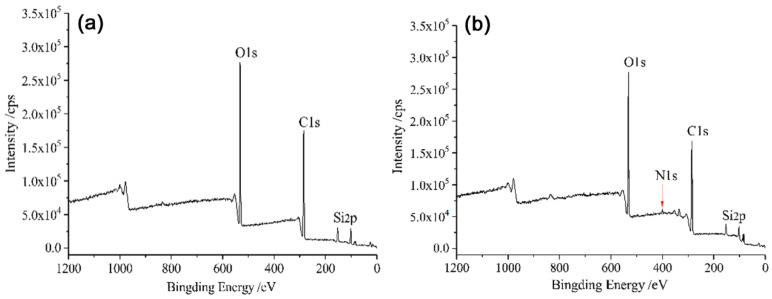
X-ray photoelectron spectroscopy (XPS) results of film for Sample 3 before (**a**) and after (**b**) bovine serum albumin (BSA) adsorption.

In order to further confirm the results of QCM-D and measure the amount of BSA adsorption of the sample films, the content of N element on the membrane surface before and after the adsorption of BSA was determined by XPS measurement. We selected Sample S3 with the best BSA resistance performance for XPS test, and the results are shown in Figure 7. The element distribution is listed in Table 6. Figure 7a shows the XPS energy spectrum of Sample S3 before BSA adsorption, and the signal peaks of carbon, oxygen, and silicon element can be clearly seen. Figure 7b shows the XPS energy spectrum of Sample S3 after the adsorption of BSA. In addition to signal peaks of carbon, oxygen, and silicon element, signal peaks of nitrogen element also appear. nitrogen element is attributed to signal peaks of BSA. This indicates that a certain amount of BSA is adsorbed on the film surface of S3. However, the signal of N1s is very weak, which indicated that only a little adsorbed BSA (N% = 1.68) (Table 6) was on the surface of film. The result of XPS proved that SiO_2_-g-PMMA-b-P(PEGMA) films have an adequate BSA resistance, which is consistent with the results of QCM-D.

### 3.5. The Thermostability of Hybrids

**Figure 8:**
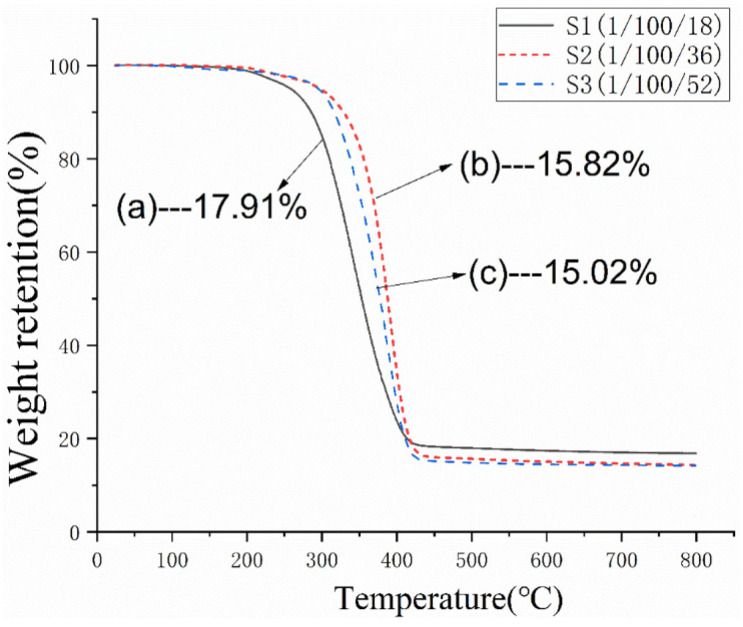
Thermo gravimetric analysis (TGA) curves of SiO_2_-g-PMMA-b-P(PEGMA) (Sample S1–S3).

The thermostabilities of obtained hybrids were determined by TGA measurement (Figure 8). As can be seen from the Figure 8, Samples S2 and S3 has similar decomposition temperature at 288 °C and similar weight loss rate is 15.82% and 15.02%wt. As Sample S1 contains the least PEGMA segments, it has a lower thermal decomposition temperature at 250 °C and a weight loss rate is 17.91% wt. This is due to P(PEGMA) segment has long PEG side chains and these side chains become entangled, which leads to an increase in the thermal decomposition temperature. The TGA analyses indicate that SiO_2_-g-PMMA-b-P(PEGMA) hybrids have an adequate thermal stability.

## 4. Conclusions

In recent work, silica-di-block polymer SiO_2_-g-PMMA-b-P(PEGMA) hybrids with three ratios (SiO_2_/MMA/PEGMA = 1/100/18, 1/100/36, and 1/100/52) were obtained by SI-ATRP method. All results of the experimental studies can be summarized as follows:

- The results of ^1^H-NMR, GPC, and TEM prove that the SiO_2_-g-PMMA-b-P(PEGMA) hybrids have been obtained as expected.
- The LSCT of hybrids is 52 °C, 56 °C, and 64 °C, respectively. The introduction of silica and hydrophobic PMMA blocks can significantly reduce LCST temperature.
- The surface of sample films presents distributed uniform granular islands. The film surface formed is relatively smooth and the surface roughness gradually decreased slightly with the growth of P(PEGMA). All films show an adequate film-forming performance and hydrophilic property.
- The QCM-D results indicate that hybrid films have an adequate BSA resistance property (△f = −18.6~−11.6 Hz). The XPS results also indicate that hybrid films have an adequate BSA resistance (N% = 1.68).
- The TGA analyses indicate that the obtained hybrids have an adequate thermal stability (288 °C).
- This paper provides a method to obtain hybrid materials with BSA resistance and film-forming properties through introducing block polymers with film-forming properties into hydrophilic copolymers by SI-ATRP.

The obtained hybrid materials have an adequate BSA resistance, LCST, and thermal stability. Thus, the SiO_2_-g-PMMA-b-P(PEGMA) hybrids could be served as BSA resistance coatings. 

The protein resistance performance of SiO_2_-g-PMMA-b-P(PEGMA) hybrids remains to be further studied and more proteins will be tested to support the statement concerning protein-resistant coatings. Further research will be conducted on the application of the coating, for example as a coating for the protection of ancient sandstone.

## Figures and Tables

**Table 1:** Condition and ingredient ratios of SiO_2_-g-P(MMA)-b-P(PEGMA).

Samples	SiO_2_-Br/MMA/PEGMA (Molar Ratios)	SiO_2_-Br (mmol)	MMA (mmol)	PEGMA (mmol)	CuCl (mmol)	CuCl_2_ (mmol)	PMDETA (mmol)	DMF (ml)
S1	(1/100/18)	0.5626	56.26	10.13	0.5626	0.0443	0.5626	20
S2	(1/100/36)	0.5626	56.26	20.25	0.5626	0.0443	0.5626	20
S3	(1/100/52)	0.5626	56.26	29.25	0.5626	0.0443	0.5626	20

**Table 2:** Original names and abbreviations to improve the readability.

Original Names	Abbreviations	Original Names	Abbreviations
methyl methacrylate	MMA	N, N, N′, N,′N″-pentamethyl diethylenetriamine	PMDETA
poly (methyl methacrylate)	PMMA	Nuclear magnetic resonance	^1^H NMR
poly (ethylene glycol) methyl ether methacrylate	PEGMA	gel permeation chromatography	GPC
poly (poly (ethylene glycol) methyl ether methacrylate)	P(PEGMA)	transmission electron microscopy	TEM
Silica nanoparticles	SiO_2_	atomic force microscope	AFM
silica-initiator	SiO_2_-Br	static contact angle	SCA
SiO_2_-Br/MMA/PEGMA (1/100/18)	S1	lower critical solution temperature	LCST
SiO_2_-Br/MMA/PEGMA (1/100/36)	S2	thermo gravimetric analysis	TGA
SiO_2_-Br/MMA/PEGMA (1/100/52)	S3	dynamic light scattering	DLS
phosphate buffer solution	PBS	Bovine serum albumin	BSA
tetrahydrofuran	THF	Quartz crystal microbalance with dissipation	QCM-D
weight-average molecular weight	Mw	numeral average molecular weight	Mn
N, N-dimethylformamide	DMF	X-Ray photoelectron spectroscopy	XPS

**Table 3:** GPC result of PMMA-b-P(PEGMA).

Samples	Mw/Mn	Mn/g·mol^−1^	Mw/g·mol^−1^
S1	1.19	15,800	18,800
S2	1.21	21,800	26,400
S3	1.25	26,600	33,000

**Table 4:** Water contact angles of sample films.

Sample	S1 (1/100/18)	S2 (1/100/36)	S3 (1/100/52)
θ_H2O_/°	35	12	-

**Table 5:** The △*f* and △*D* data for the sample films casted by THF solution.

Samples	△*f* (Hz)	△*D*(10^−6^)
S1(1/100/18)	18.6	0.30
S2(1/100/36)	15.4	0.55
S3(1/100/52)	11.6	1.30

**Table 6:** The chemical composition of film for Sample S3.

Samples	C (%)	O (%)	Si (%)	N (%)
S3(before)	59.67	32.21	8.12	-
S3(after)	56.51	33.56	8.07	1.86

(where, before in brackets refers to before BSA adsorption and after refers to after protein adsorption)
